# Paretic versus non-paretic stepping responses following pelvis perturbations in walking chronic-stage stroke survivors

**DOI:** 10.1186/s12984-017-0317-z

**Published:** 2017-10-13

**Authors:** Juliet A. M. Haarman, Mark Vlutters, Richelle A. C. M. Olde Keizer, Edwin H. F. van Asseldonk, Jaap H. Buurke, Jasper Reenalda, Johan S. Rietman, Herman van der Kooij

**Affiliations:** 1 Roessingh Research and Development, Enschede, The Netherlands; 2 Department of Biomechanical Engineering, University of Twente, Horstring W119, PO Box 217, 7500 AE Enschede, The Netherlands

**Keywords:** Stroke, Balance during gait, Perturbed walking, Reactive foot placement, Muscle activity changes

## Abstract

**Background:**

The effects of a stroke, such as hemiparesis, can severely hamper the ability to walk and to maintain balance during gait. Providing support to stroke survivors through a robotic exoskeleton, either to provide training or daily-life support, requires an understanding of the balance impairments that result from a stroke. Here, we investigate the differences between the paretic and non-paretic leg in making recovery steps to restore balance following a disturbance during walking.

**Methods:**

We perturbed 10 chronic-stage stroke survivors during walking using mediolateral perturbations of various amplitudes. Kinematic data as well as gluteus medius muscle activity levels during the first recovery step were recorded and analyzed.

**Results:**

The results show that this group of subjects is able to modulate foot placement in response to the perturbations regardless of the leg being paretic or not. Modulation in gluteus medius activity with the various perturbations is in line with this observation. In general, the foot of the paretic leg was laterally placed further away from the center of mass than that of the non-paretic leg, while subjects spent more time standing on the non-paretic leg.

**Conclusions:**

The findings suggest that, though stroke-related gait characteristics are present, the modulation with the various perturbations remains unaffected. This might be because all subjects were only mildly impaired, or because these stepping responses partly occur through involuntary pathways which remain unaffected by the complications after the stroke.

## Background

Stroke survivors often experience problems with maintaining their balance. A variety of neurological deficits can hamper balance control during walking, such as hemiparesis, sensory impairments, as well as cognitive problems such as fear of falling. As a consequence, fall rates in stroke survivors are 2–8 times higher than those in healthy, age-matched subjects [1]. In general, especially balance control in the frontal plane is often considered challenging, requiring adequate foot placement to continue walking [2]. This might be an additional challenge when suffering from hemiparesis following a stroke, which could lead to differences in recovery steps made with the paretic and the non-paretic leg in response to a disturbance during walking. To reduce the fall risk of stroke survivors and make their rehabilitation more effective, it is required to characterize how balance control is affected by a stroke. Such knowledge might be used to provide limb-specific support in robot-assisted gait rehabilitation.

Stroke survivors typically show differences in gait characteristics between the paretic and non-paretic leg during unperturbed walking, for example as a result of decreased motor control in the paretic leg. In a study by Balasubramanian et al. subjects placed the paretic leg at an increased lateral distance from the pelvis compared to the non-paretic leg in mediolateral (ML) foot placement during unperturbed walking [3]. However, no differences in step width were found with regard to the leg used for stepping. It is therefore of importance to consider both legs individually, in a body referenced frame such as that of the center of mass (COM). Dean et al. studied the relation between gluteus medius muscle activity in the swing leg and both the ML position and velocity of the COM relative to the stance foot [4]. For low fall-risk subjects the results suggest a stronger activity modulation in the non-paretic swing leg than in the paretic swing leg, though it did not show how both legs respond to actual destabilizing conditions such as external perturbations.

Perturbations can be used to affect the body state, such as the position and velocity of the COM relative to the stance foot. This may lead to adjustments in foot placement location and timing to maintain balance. In Krasovsky et al. perturbations were applied by unexpectedly arresting the ankle of the leg at early swing [5]. Stroke survivors showed shorter step lengths and shorter swing times compared to healthy controls when stepping in response to perturbations applied to the non-paretic swing leg. Furthermore, in Hak et al. continuous ML support surface translations were used to assess if and how low-fall risk stroke survivors change their base of support (BoS) during walking through foot placement adjustments [6]. It was found that stroke survivors more strongly shortened their step length and step time, and increased their step width, as compared to healthy controls. However these studies did not compare the perturbation responses of the individual paretic and non-paretic limbs.

In healthy subjects, ML foot placement adjustments in response to ML perturbations relate to the ML COM velocity [7, 8]. This is reflected in a concept called the extrapolated center of mass (XCOM), which has been previously used to indicate fall risk in relation to foot placement [6, 9]. The XCOM can be derived from a linear inverted pendulum model [10, 11] and can be regarded as a point on the floor at a horizontal distance from the COM. This distance is directly proportional to the horizontal COM velocity through a proportionality constant *ω*_*0*_^*−1*^. If the inverted pendulum model would place its point-foot onto the XCOM, the model will stop moving in an upright position. Placing the foot further away from the COM than the XCOM will cause the model to fall back in the direction from which it came. The further the model places its foot beyond the XCOM, the sooner the model is redirected in the opposite direction. Such movement is relevant to the ML direction of walking, where toppling over the leg is often not desired. Hence, investigating paretic and non-paretic foot placement in relation to the XCOM allows analysis from a simple model perspective.

The main purpose of this study is to characterize differences between the paretic and the non-paretic legs in walking chronic-stage stroke survivors in terms of ML stepping following ML pelvis perturbations. Rather than applying continuous disturbances, we will use transient perturbations directed inward and outward. We will also investigate how the steps are made in relation to the XCOM. Only low fall-risk subjects will be considered, to reduce the risk of actual falls in response to the perturbations.

## Methods

### Participants

Ten stroke survivors in the chronic stage (7 male, age: 52 ± 16 years, weight: 82.5 ± 13.6 kg, height: 1.75 ± 0.06 m, mean ± std) were recruited. All gave written informed consent before participation. All participants had a Functional Ambulatory Category score of 4, meaning that these subjects can walk independently on even surfaces, but might require assistance in more challenging situations such as uneven terrain [12]. Related clinical measures can be found in Table 1. The experimental setup and protocol were approved by the local ethics committee, and was in accordance with the Declaration of Helsinki.

**Table 1:** Subject Characteristics

ID	Gender (M/F)	Age (yrs.)	Time post-stroke (yrs.)	Affected leg (L/R)	Weight Distr. Stance (%) (nonPar/Par)	Weight Distr. Walking (%) (nonPar/Par)	BBS (pts)	DGI (pts)	MMSE (pts)	FES-I (pts)	10MWT (km/h)	Treadmill speed (km/h)
1	M	66	10	R	54/46	58/42	51	–	28	16	–	0.4
2	M	30	7	R	48/52	56/44	55	22	30	30	4.5	1.0
3	F	70	9	R	51/49	59/41	55	22	26	17	1.9	0.7
4	M	63	10	L	54/46	54/46	55	22	27	19	4.6	2.0
5	M	38	5	R	40/60	50/50	48	21	28	29	3.3	1.6
6	M	67	7	L	55/45	57/43	53	–	30	22	–	2.2
7	M	64	4	L	56/44	54/46	56	–	26	16	–	5.0
8	M	45	3	R	46/54	43/57	56	23	27	34	4.7	2.2
9	F	29	8	L	54/46	61/39	56	22	28	17	3.7	2.2
10	F	46	29	L	61/39	58/42	56	24	30	17	4.7	2.4
Mean (±std)	7 M,3F	52 (±16)	9 (±7)	5R,5 L	52/48 (±6)	55/45 (±5)	54 (±3)	22 (±1)	28 (±2)	22 (±7)	3.9 (±1.0)	2.0 (±1.3)

*BBS* Berg Balance Score*, DGI* Dynamic Gait Index*, MMSE* Mini-Mental State Examination*, FES-I* Falls Efficacy Scale International*, 10MWT* 10 Meter Walking Test

### Apparatus

**Fig. 1:**
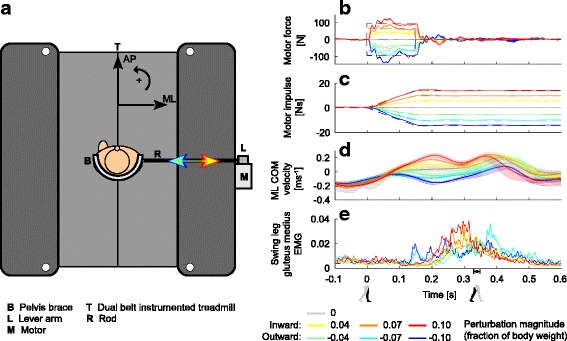
Experimental setup and single-subject perturbation profiles. **a** Schematic overview of the experimental setup. In the schematic the left leg is the stance leg. For the right stance leg the leftward and rightward arrow colors interchange. **b** Reference (dashed) and measured (solid) interaction force between motor and subject. **c** Reference (dashed) and measured (solid) motor impulse, obtained by integrating the interaction forces. **d** ML COM velocity relative to the walking surface. **e** EMG profile of the swing leg gluteus medius. Colors indicate the various perturbations. Lines represent within-subject averages of a single subject. Shaded areas indicate the within-subject standard deviation. The subject shown was the subject with the highest step frequency among subjects. The square on the time axis indicates the average time of heel strike after perturbation onset at toe-off (paretic and non-paretic pooled)

Subjects walked on an instrumented treadmill (custom Y-mill, Motekforce Link, Culemborg, the Netherlands), used to collect 3 degrees-of-freedom ground reaction forces and torques per foot. Subjects could be perturbed in the ML direction using a motor (SMH60, Moog, Nieuw-Vennep, the Netherlands) bolted to a support structure clamped to the treadmill’s exterior frame. A brace (Distrac Wellcare, Hoegaarden, Belgium) worn by the subject could be connected to the motor through an aluminum horizontal rod and a vertical lever arm. The rod was connected to both the brace and the lever arm through ball joints, to allow freedom of movement in the anteroposterior (AP) direction. Given the motor range of motion, the maximum lateral excursion from the center of the treadmill was approximately 0.55 m in each direction. The motor was admittance controlled over Ethernet (User Datagram Protocol) at 1000 Hz, using xPC-target (The Mathworks, Natick, US). A schematic overview of the experimental setup can be found in Fig. 1a. Additional details can be found in Vlutters et al. [8].

### Data collection

Subject kinematic data were collected at 100 Hz using a 9 camera motion capture system (Visualeyez II, Phoenix Technologies Inc., Burnaby, Canada). Marker clusters, each consisting of 3 LEDs, were placed on the feet, lower legs, upper legs, pelvis, sternum and head. Additional single LEDs were placed on both lateral malleoli, and both lateral epicondyles of the femur. Gluteus medius EMG activity in both legs was recorded using a Bagnoli Desktop EMG system (Delsys, Natick, MA, USA) at 1000 Hz. Finally, subjects were filmed from the rear using a video camera to be able to detect any instances during which the subject grasped the handrails of the treadmill.

### Protocol

Various bony landmarks were indicated using an LED based probe [13], prior to the experiment. These landmarks were the 1st and 5th metatarsal heads, calcaneus, medial and lateral malleoli, fibula head, medial and lateral epicondyles of the femur, greater trochanter of the femur, anterior and posterior superior iliac spines, xiphoid process, jugular notch, 7th cervical vertebra, occiput, head vertex and nasal sellion [14]. Subjects wore a safety harness at all times to prevent injury in case of a fall. The preferred walking speed was determined by gradually increasing the speed of the treadmill, until the subject felt comfortable. This was also used to have subjects familiarize with walking on the treadmill.

During the experiment subjects wore the brace around the pelvis and were instructed to walk at the center of the treadmill. First, a 2 min baseline measurement at the preferred walking speed was recorded without applying perturbations. When no perturbations were applied the motors were admittance controlled such that the interaction force between the subject and the motor was as low as possible, see Fig. 1b. In subsequent trials, perturbations could be randomly applied at the instance of toe-off of either leg. This way subjects were required to use both the paretic and the non-paretic leg in the first recovery step. The toe-off instances were detected using the vertical ground reaction forces. Perturbations were 150 ms block pulses of magnitudes equal to 4, 7, and 10% of the subject’s body weight. The direction could be inward (e.g. leftward for left stance) or outward (e.g. rightward for left stance). Though the motors cannot track the desired reference force, the delivered impulse is approximately as desired, see Fig. 1c. Each perturbation condition was repeated 5 times, yielding 60 perturbations per subject. The perturbations were randomized over magnitude, direction, and onset instance (paretic or non-paretic toe-off). The minimum time between successive perturbations was also randomized, and varied between 6 and 12 s. Finally, Berg Balance Scale (BBS), Dynamic Gait Index (DGI), Mini-Mental Scale Examination (MMSE), Fall Efficacy Scale International (FES-I) and 10-m-walking-test (10MWT) measures were collected within 1 week after the experiment.

### Data processing

Data were processed using Matlab (R2013b, Mathworks, Natick, US). EMG data were detrended, filtered with a 1st order 48–52 Hz Butterworth bandstop filter, then rectified, and filtered with a 1st order 20 Hz Butterworth low-pass filter. Marker data were filtered with a 4th order 20 Hz zero-phase Butterworth low-pass filter before reconstructing the global bony landmark positions from the probe measurements. Using the landmarks, the COM positions of the captured segments were estimated following Dumas et al. [14]. The total body COM was calculated as a weighted average of the segment COM positions, and was differentiated for a COM velocity. All other position and velocity data were expressed relative to those of the COM. Gait events of toe-off and heel strike were detected using landmarks of the feet [15]. Instances of perturbation onset were detected from the motor input signals. The recorded video data were visually inspected for perturbations during which subjects touched the hand-rails of the treadmill. Corresponding trials were removed from analysis.

For each subject, the position of the COM of both feet relative to the total-body COM, as well as the COM velocity, were extracted at the instances of heel strike and toe-off after the perturbation. The gait phase durations between these instants were also calculated. The EMG data of each individual muscle was scaled to the median of the maximum values occurring every gait cycle during the unperturbed walking condition. For each subject and in each condition, the scaled EMG time-averages between toe-off and heel strike were calculated, representing the averaged muscle activity level during the swing phase. All these data were sorted on perturbation magnitude, perturbation direction (inward or outward), and leg used for stepping (paretic or non-paretic). Since five right-sided and five left-sided paretic subjects participated in the study, all position, velocity, and EMG data resulting from the left side of the body were laterally mirrored for all subjects. All data in this paper is therefore presented as if the first recovery step was made with the right leg, for both the paretic and the non-paretic leg of all subjects. Next, for each subject all data were averaged over the repeated conditions to obtain repetition averages. These were in turn averaged for subject-averages and standard deviations. Finally, for each subject an XCOM proportionality constant was calculated following *ω*_*0*_^*−1*^ = √(*l*/*g*) [7], in which *l* is the subject’s leg length, and *g* the earth’s gravitational acceleration. These ω_0_^−1^ were averaged across subjects for a subject-average proportionality constant.

### Statistical analysis

Linear mixed models were used to determine the effect of all perturbations (fixed factor, with intercept), the effect of the leg used in the first recovery step (fixed factor, with intercept), and their interaction effect on the outcome measures. Outcome measures were the ML and AP distance between the leading foot and the COM, the ML and AP distance between both feet (step width and step length, respectively), the ML COM velocity at heel strike, the gait phase durations following the perturbations, and the EMG signal averages. To account for correlated repeated measures within the same subject, a random subject factor (intercept) was included in the model. The significance level was set to α = 0.05, and a Dunn-Šidác correction was applied during post hoc analysis. In the post hoc comparison, perturbation data were only compared to the unperturbed condition and not mutually. The Statistical Package for the Social Sciences version 19.0 (IBM Corporation, Armonk, NY, USA) was used for the statistical analysis.

## Results

All subjects completed the experiment without falling. Subjects 1, 6, and 7 did not complete the DGI and the 10MWT. Subjects 1, 2, and 3 grabbed the handrails 4, 4, and 6 times respectively, mainly in response to the largest magnitude inward perturbations. The corresponding data were removed from analysis. Subject characteristics are presented in Table 1.

### Foot placement location in ML and AP directions

**Fig. 2:**
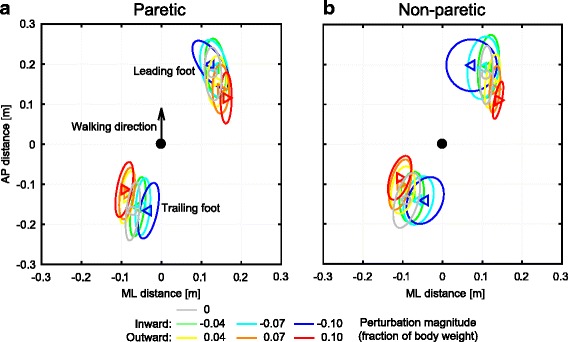
Positions of the COM of the feet relative to the whole body COM. **a** Locations of the COM of both the leading and the trailing foot relative to the whole-body COM, at the instant of heel strike, for steps made with the paretic leg. **b** Same as in A, for steps made with the non-paretic leg. Triangles show subject averages and indicate the perturbation direction. Ellipses represent subject standard deviations. Colors indicate the perturbation magnitudes as a fraction of the subject’s body weight

Perturbations resulted in foot placement modulation. Here, we specifically focus on the location of the leading foot. Perturbations were applied such that each subject had to make the first recovery step with both the paretic- and the non-paretic leg. The locations of the feet relative to the COM at the instant of the first heel strike after the perturbation are presented in Fig. 2. Note that because the data is presented relative to the COM, the stance foot data changes with the perturbations because the body is pushed away from it or towards it. Foot placement relative to the COM as a result of the different perturbations was not found to differ between the paretic and non-paretic leg, for both ML (F_6,117_ = 0.981, *p* = 0.441) and AP (F_6,117_ = 0.068, *p* = 0.999) distances between the COM and the leading foot. This indicates that no interaction effect was present between factors perturbation and leg used for stepping.

When considering the effects of the leg used in the stepping response, it is important to regard the foot location relative to the COM rather than relative to the trailing foot. In the frontal plane, the foot of the paretic leg is always laterally placed further away from the COM than the foot of the non-paretic leg (F_1,123_ = 26.956, *p* < 0.001). Instead, when considering step width, i.e. the ML the distance between the feet, no significant differences were found between the legs used in the first recovery step (F_1,123_ = 2.086, *p* = 0.151). For the AP distance between the COM and the leading foot no significant effects of factor leg were found (F_1,123_ < 0.001, *p* = 0.999). However the step length, i.e. the AP distance between the feet, was larger when stepping with the paretic leg compared to stepping with the non-paretic leg (F_1,123_ = 9.294, *p* = 0.003). The latter effects are therefore caused by the trailing leg, and are likely related to the time spent standing on that leg.

Mediolateral adjustments with the leading foot mostly occur in the direction of the perturbation. Especially for outward perturbations the foot appears to be placed further in the direction of the perturbation with increasing perturbation magnitude. Significant effects of factor perturbation were found for the ML distance between the COM and the leading foot (F_6,123_ = 7.215, *p* < 0.001), as well as for the AP distance between the COM and the leading foot (F_6,123_ = 24.799, *p* < 0.001). Post hoc analysis revealed that this AP distance decreased significantly for all outward perturbation compared to unperturbed walking (*p* < 0.002), but did not change significantly for all inward perturbations (*p* > 0.160).

### Relation between ML foot placement and ML COM velocity

**Fig. 3:**
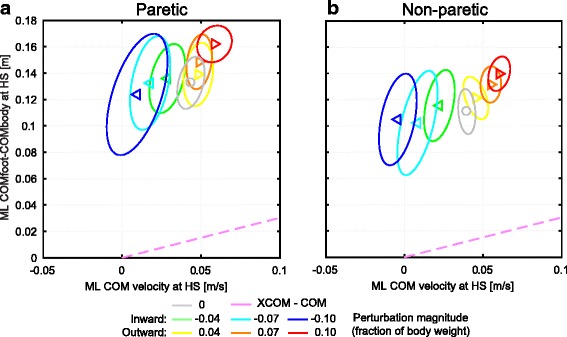
Distance between the COM of the leading foot and the whole body COM against the COM velocity. **a** For recovery steps made with the paretic leg, at the instant of the first heel strike after the perturbation. **b** Same as in A, but for steps made with the non-paretic leg. Triangles show subject averages and indicate the perturbation direction. Ellipses represent subject standard deviations. Colors indicate the perturbation magnitudes. The pink dashed line indicates the position of the XCOM relative to the COM. This line has a slope of ω_0_^−1^ and no intercept

We investigated the mediolateral stepping location in relation to the ML COM velocity at the instant of heel strike, specifically in relation to the location of the XCOM at heel strike, see Fig. 3. If the ML distance between the COM and the leading foot (y-axis) would increase with increasing mediolateral velocity (x-axis) at the same rate (ω_0_^−1^) as that of the pink XCOM line, then the foot would be placed at a fixed distance from the XCOM [7]. This would provide a simple explanation for lateral foot placement, based on a concept derived from a linear inverted pendulum model.

Factors perturbation and leg had a significant interaction effect on the ML COM velocity following the perturbations (F_6,114.068_ = 2.587, *p* = 0.022). As a result, steps made with the non-paretic leg show more variability in the COM velocity between the various perturbations, as compared to that of steps with the paretic leg. That is, for steps with the non-paretic leg outward perturbations lead to more positive COM velocities, whereas for inward perturbations it leads to smaller, or even a slightly negative COM velocity. Factor leg also provided a significant main effect on the ML COM velocity, with a higher velocity for steps made with the non-paretic leg (F_1,114.058_ = 7.011, *p* = 0.009). Furthermore, the various perturbations significantly affected the ML COM velocity at heel strike (F_6,114.068_ = 82.830, *p* < 0.001), increasing in the direction of the perturbation with increasing perturbation magnitude (post hoc: step with paretic: *p* ≤ 0.027 for all perturbations except for 0.04, 0.07 outward, step with non-paretic: *p* ≤ 0.007 for all perturbations except for 0.04 outward). While at heel strike the ML distance between the COM and the leading foot showed modulation with the COM velocity, the average distances do not appear to modulate with the COM velocity in the same way as the XCOM does. That is, the ML distance of the foot relative to the COM does not relate to the ML COM velocity through a factor *ω*_*0*_^*−1*^. Instead, both the paretic and the non-paretic leg show an increase in foot-COM distance with increasing outward perturbation magnitude, but remain approximately the same with increasing inward perturbation magnitude. Therefore, for the presented results the XCOM alone is not a sufficient predictor of the lateral stepping location, though this might be different in a more homogeneous subject group.

### Gait phase durations

For all presented gait phase durations, modulation with the perturbations is invariant of the leg with which the step is made. No significant interaction effects were found between factors perturbation and leg on the duration of the swing phase in which the perturbation was applied (F_6,117_ = 0.163, *p* = 0.986), nor on the subsequent double support phase duration (F_6,115.993_ = 1.045, *p* = 0.400).

**Fig. 4:**
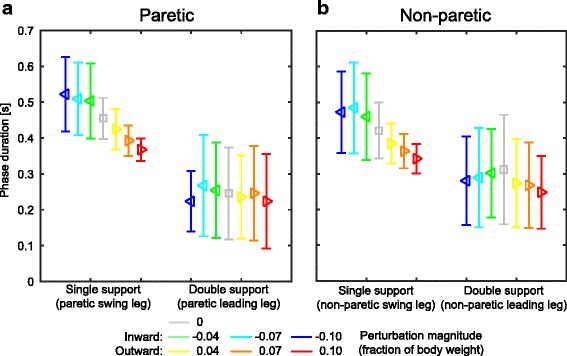
Gait phase durations. **a** The single support duration during which the perturbations were applied, and the subsequent double support duration, for recovery steps made with the paretic leg. **b** Same as in A, but for recovery steps made with the non-paretic leg. Triangles show subject averages and indicate the perturbation direction. Vertical bars represent subject standard deviations. Colors indicate the perturbation magnitudes

The most prominent gait phase duration modulation occurred in the swing phase during which the perturbation was applied. The duration of this swing was significantly affected by the perturbations (F_6,123_ = 26.035, *p* ≤ 0.001), significantly increasing with increasing inward perturbation magnitude (*p* ≤ 0.045), and decreasing with increasing outward magnitude (*p* ≤ 0.002, except 0.04 outward), see Fig. 4. In contrast, the subsequent double support durations were not significantly affected by factor perturbation (F_6,121.993_ = 1.864, *p* = 0.092).

The gait phase durations also reflect the differences between steps made with the paretic and the non-paretic leg. Subjects preferred to spend more time on the non-paretic leg than on the paretic leg during the single support phase, regardless of which perturbation was applied (F_1,123_ = 16.448, *p* ≤ 0.001). Furthermore, the double support phase during which the weight is shifted from the paretic leg toward the non-paretic leg also last longer compared to the opposite shift (F_1,121.993_ = 23.365, *p* ≤ 0.001).

### Gluteus medius activity

Subjects modulated the gluteus medius activity in the swing leg with the various perturbations, for both the paretic and non-paretic leg. Though more varied modulation appears to occur in the non-paretic leg over all perturbations, no interaction effects were found between factor leg and perturbation magnitude (F_6,88.978_, *p* = 0.452).

**Fig. 5:**
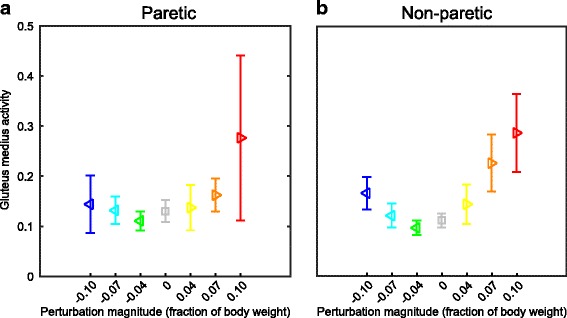
EMG responses. **a** For the paretic leg, modulation with the perturbation magnitude of the time-average EMG signals between toe-off (perturbation onset) and heel strike. **b** Same as in A, but for the non-paretic leg. Triangles show subject averages and indicate the perturbation direction. Vertical bars represent subject standard deviations. Colors indicate the perturbation magnitudes

However, a significant effect of the perturbations was found on the gluteus medius activity levels (F_6,94.917_ = 18.993, *p* < 0.001), increasing on average. Especially for outward perturbations where the gluteus medius participates in foot placement adjustments through leg abduction, increased activity levels can be observed with increasing perturbation magnitude (*p* < 0.001, except for 0.04 outward), see Fig. 5. For inward perturbations the post hoc comparison provides no significant effects, though the non-paretic swing leg appears to show more EMG modulation than the paretic swing leg. Differences between both legs were not tested as activity levels of both legs were scaled to baseline values of each leg.

## Discussion

We investigated the first recovery step following mediolateral pelvis perturbations in chronic-stage stroke survivors who walked at a self-selected speed. The transient perturbation were randomly applied at the instant of toe-off of the paretic and non-paretic leg, such that every subject had to make recovery steps with both legs. The aim was to evaluate and compare stepping responses regarding the use of the paretic and non-paretic leg. In accordance to our hypothesis, we found various differences in gait characteristics between steps made with the paretic and non-paretic leg. However, foot placement modulation in response to the various perturbations did not differ between the legs within the included group of stroke survivors.

### Foot placement with respect to the COM

Our findings in perturbed walking support the analysis of foot placement measures with respect to the COM, similar as previously applied in unperturbed walking [3]. As in the work by Balasubramanian et al., in the current results the mediolateral distance between the feet is independent of the leg with which the step is made. However, this does not imply that there are no differences between both legs in terms of foot placement, as the paretic leading leg is placed at a consistent increased lateral distance from the body’s COM. It has been suggested that this might be related to strength limitations in the paretic leg [3, 16], leading to the typical weight bearing asymmetry during stance [17]. These limitations could in turn lead to limited control over the COM motion with the paretic leg [18, 19], especially during the single support phase in which the paretic leg is the stance leg. This might also explain the larger variability in ML COM velocity following the perturbations applied during the paretic single support phase compared to those during the non-paretic single support phase. The results suggest that classical gait parameters such as step length and width might not fully capture balance and walking impairments in stroke survivors. Analysis of the feet locations relative to the COM might identify leg specific aspects of the impairment, and help specify gait-phase specific support in robotic gait training and rehabilitation.

### Leg-independent step modulation in response to perturbations

The results imply that subjects attempt to execute similar stepping strategies with both legs, and that the modulation with the various perturbations is not hampered by the effects of the stroke. Though the paretic leg is laterally placed further away from the COM than the non-paretic leg when making a step, the way in which the foot placement location alters with the various perturbations does not statistically differ between both legs. Consequently, subjects do not modulate their stepping location worse with the paretic leg than with the non-paretic leg, in response to the various perturbations. Similar effects were observed in modulation of the gait phase duration following the perturbations. Although subjects spent more time standing on the non-paretic leg, they showed comparable changes in gait phase durations with both the paretic and the non-paretic legs in response to the different perturbations. One potential cause might be that the subjects were only mildly affected by the stroke, as classified by their clinical scales. Subjects with lesser scores on clinical outcome measures might have shown larger deviations. For instance, in a study by Dean et al. high fall risk subjects showed different stepping strategies compared to low fall risk subjects [4]. Another potential reason might be that the modulation is partly automatic. For example, Nashner et al. showed that balance perturbations during standing can trigger rapid postural adjustments that take precedence over voluntary movements [20]. It might be that these responses involve neural pathways that bypass the brain areas damaged by the stroke.

### Gluteus medius activity modulation

It has previously been suggested that swing leg gluteus medius activation for ML foot placement might be based on sensory information from the stance leg [4, 21]. Our outward perturbations move the COM further away from the stance foot with increasing perturbation magnitude. Therefore, as in Dean et al., gluteus medius activity increased with increasing ML distance between the COM and the stance foot [4]. These effects occurred in both the paretic and the non-paretic swing legs, without significant interaction effects between factors leg and perturbation. These findings therefore match those for step location and step time modulation in this study, suggesting that modulation with the perturbations is not different between both legs in the current subject group.

With the current setup in stroke survivors, it is not possible to confirm whether this relation between the stance leg and the gluteus medius activity of the swing leg is a causal one. As stroke survivors often suffer from sensory impairments in the paretic leg [22], one would expect information from the stance leg to be less reliable if that leg is paretic. Consequently, if gluteus medius activity modulation would primarily depend on the state of the stance leg, one would expect the paretic swing leg to receive the best information. However, in Dean et al. the gluteus medius of the paretic swing leg showed poorer modulation with the state of the stance leg than that in the non-paretic swing leg [4]. It is therefore likely that effects of the stroke on the paretic leg dominate the relations in Dean et al., rather than how well information is conveyed from one leg to the other.

### Absence of cross-stepping

In a similar study with healthy subjects, inward perturbations of sufficient magnitude lead to cross-steps [7]. These were not observed in the current study. Though the maximum perturbation magnitudes were not as high as in Vlutters et al. [8], subjects visually appeared to explicitly avoid crossing the legs. In case of larger magnitude inward perturbations, subjects tended to move the arms, or abduct the swing leg, possibly using angular momentum strategies [23, 24] to recover from the perturbations. Similar leg abduction responses were reported in Dean et al. for unperturbed high fall-risk subjects [4]. Subjects might be reluctant to make cross-steps, as entangling the legs would hamper gait progression. These effects might be amplified by walking on a treadmill, as it requires the subjects to keep walking to prevent falling off the belt.

### Analysis from a model perspective

Evaluating foot placement in relation to the XCOM as derived from a linear inverted pendulum model might aid in understanding why a subject places the foot at a certain location. It can be deducted how such a model would move depending on where its foot is placed relative to the XCOM. In general, subjects laterally placed their foot beyond the XCOM, further away from the COM than the XCOM itself. The foot was placed further beyond the XCOM for steps made with the paretic leg as compared to steps made with the non-paretic leg. Doing so in the model would make the COM fall back toward the other leg, but at a higher rate for steps made with the paretic leg. This would require the subsequent step with the non-paretic leg to be of shorter duration, therefore spending less time standing on the paretic leg. This is in line with our findings, as well as with other studies in unperturbed walking [25, 26].

Previous studies have investigated the XCOM as a linear predictor for a mediolateral stepping location in healthy subjects [7, 8]. In Vlutters et al., the COM velocity related linearly to the mediolateral stepping location after the perturbation [8]. While the ML stepping location in our stroke subjects does modulate with COM velocity, and therefore with the XCOM, the data shows substantial variability. The strong linear trend observed in healthy subjects does therefore not hold here. This implies that the COM velocity holds some predictive power for a mediolateral stepping location in this group of stroke survivors, but not as apparent as in healthy subjects, or perhaps not through a linear relation.

### Effects of walking speed

The subjects in this study were allowed to walk at a self-selected comfortable walking speed, resulting in a treadmill speed range from 0.4 to 5.0 km/h among subjects. This range leads to variability in spatio-temporal gait parameters such as step length, width, and frequency, which all alter with gait speed. However, our findings focus on the differences between the paretic and the non-paretic legs of the subject population as a whole. The effects of walking speed are likely to be equal for both legs within a single subject, allowing comparison between both legs throughout the subject population.

### Limitations

The perturbation device was controlled such that the interaction force between the subject and the motor was as low as possible. The total reflected inertia from the motor to the pelvis is about 1 kg. In a study by Meuleman et al. it was concluded that up to 5.3 kg of inertia can be added to mediolateral pelvis movement without significantly affecting the gait [27]. Interaction with the motor is therefore not expected to affect the subjects’ gait when no perturbation is applied. However, the rod used to connect the motor to the pelvis brace partly obstructed the right arm sway. Subjects were not able to fully swing their arm backward during the measurement trials, thereby possibly affecting the gait kinematics. Several subjects indicated that the rod was not a problem, or preferred walking with their arms in front of their body to hold the paretic hand. Little effects of this constraint on the lower limb responses are therefore expected.

It has previously been shown that self-selected overground walking speed as assessed by the 10MWT relates to the degree of walking impairment [28]. Based on this classification, all subjects in our study are within a group of community walkers, and are mildly affected. The subject data shows that the gait speed on the treadmill is often lower than that recorded in the 10MWT. Even though subjects were given a period of time to familiarize with the measurement set-up, subjects might adapt their gait while walking on a treadmill [29] due to being unfamiliar with the measurement setup, or due to fear of falling. However, in Zadravec et al. it was demonstrated that responses of healthy subjects to horizontal pelvis perturbations show high degrees of similarity between overground and treadmill walking [30]. Hence, despite the differences in treadmill and overground walking speed in our subjects, the responses to perturbations are expected to be similar in both scenario’s given a walking speed.

Finally, the variety between subject characteristics such as the walking speed results in more variable data than what would be obtained with a more homogeneous subject group, or with more subjects. The variability in the data could affect the interpretation of the results, such as whether or not the XCOM provides predictive value for lateral foot placement in stroke survivors. Studying more consistent subject groups could reveal to what extent these relations hold for reactive stepping in response to perturbations.

## Conclusions

The current work investigated paretic and non-paretic mediolateral stepping responses following ML pelvis perturbations in walking stroke survivors. Stepping responses were investigated for the first step taken after the perturbation. Following a perturbation, subjects preferred to spend more time standing on their non-paretic leg. Analysis in a body-referenced frame identified that the paretic leading leg was placed at a consistently increased ML distance from the body’s COM. Despite these differences in gait characteristics, our results suggest that the modulation of the paretic and non-paretic leg with the various perturbations is not greatly hampered by the effects of the stroke. Foot placement and gluteus medius EMG activity imply that subjects attempted to execute similar stepping strategies with both legs. This might be explained because all subjects were classified as having low-fall risk, or because the responses might be partially involuntary, with the modulation not directly affected by the effects of stroke. Though modulation occurred in both legs, mediolateral foot placement in the presented group of stroke survivors could not be predicted using a linear predictor based on the COM velocity. If the subject is able to modulate steps using the paretic leg one might refrain from assisting leg swing during training using a robotic device or exoskeleton, and only provide support after foot contact in weight bearing.

## Abbreviations

10MWT: 10-meter-walking-test
AP: Anterior-posterior
BBS: Berg balance scale
BoS: Base of support
COM: Center of mass
DGI: Dynamic gait index
FES-I: Fall efficacy scale international
ML: Mediolateral
MMSE: Mini-mental scale examination
XCOM: Extrapolated center of mass
